# Anti-amyloid Compounds Inhibit α-Synuclein Aggregation Induced by Protein Misfolding Cyclic Amplification (PMCA)*

**DOI:** 10.1074/jbc.M113.542340

**Published:** 2014-02-28

**Authors:** Maria Eugenia Herva, Shahin Zibaee, Graham Fraser, Roger A. Barker, Michel Goedert, Maria Grazia Spillantini

**Affiliations:** From the ‡ John Van Geest Centre for Brain Repair, E. D. Adrian Building, Robinson Way, Cambridge CB2 0PY, United Kingdom and; the § Medical Research Council Laboratory of Molecular Biology, Francis Crick Avenue, Cambridge Biomedical Campus, Cambridge CB2 0QH, United Kingdom

**Keywords:** α-Synuclein, Amyloid, Cell Therapy, Drug Screening, Protein Aggregation, PMCA

## Abstract

Filaments made of α-synuclein form the characteristic Lewy pathology in Parkinson and other diseases. The formation of α-synuclein filaments can be reproduced *in vitro* by incubation of recombinant protein, but the filament growth is very slow and highly variable and so unsuitable for fast high throughput anti-aggregation drug screening. To overcome this obstacle we have investigated whether the protein misfolding cyclic amplification (PMCA) technique, used for fast amplification of prion protein aggregates, could be adapted for growing α-synuclein aggregates and thus suitable for screening of drugs to affect α-synuclein aggregation for the treatment of the yet incurable α-synucleinopathies. Circular dichroism, electron microscopy, and native and SDS-polyacrylamide gels were used to demonstrate α-synuclein aggregate formation by PMCA, and the strain imprint of the α-synuclein fibrils was studied by proteinase K digestion. We also demonstrated that α-synuclein fibrils are able to seed new α-synuclein PMCA reactions and to enter and aggregate in cells in culture. In particular, we have generated a line of “chronically infected” cells, which transmit α-synuclein aggregates even after multiple passages. To evaluate the sensitivity of the PMCA system as an α-synuclein anti-aggregating drug screening assay a panel of 10 drugs was tested. Anti-amyloid compounds proved efficient in inhibiting α-synuclein fibril formation induced by PMCA. Our results show that α-synuclein PMCA is a fast and reproducible system that could be used as a high throughput screening method for finding new α-synuclein anti-aggregating compounds.

## Teaser

**Background:** α-Synuclein filaments can be formed *in vitro*, but the process is slow and unreliable, unsuitable for high throughput screening.

**Results:** Protein misfolding cyclic amplification (PMCA) rapidly assembles α-synuclein into filaments that form aggregates in cell culture and whose formation is inhibited by anti-amyloid compounds.

**Conclusion:** Anti-amyloid compounds affect PMCA cell-transmissible α-synuclein fibril formation.

**Significance:** α-Synuclein PMCA is useful for the screening of anti-aggregation compounds as treatments for α-synucleinopathies.

## Introduction

α-Synucleinopathies are characterized by the misfolding and aggregation of the abundant CNS protein α-synuclein that is expressed predominantly in nerve cells and is concentrated at presynaptic nerve terminals, where it plays a role in synaptic vesicle transport (1). Aggregated α-synuclein forms the neuronal inclusions of Parkinson disease and dementia with Lewy bodies (Lewy bodies and neurites) and the oligodendroglial inclusions of multiple system atrophy (glial cytoplasmic inclusions) (1). Evidence that α-synuclein aggregation causes these diseases has accumulated through a variety of neuropathological, biochemical, and genetic studies (2, 4). In particular, dominantly inherited mutations and duplications and triplications of *SNCA*, the α-synuclein gene, cause familial forms of Parkinson disease, dementia with Lewy bodies, and multiple system atrophy (5), and α-synuclein has been identified as a risk factor for Parkinson disease in all genome-wide association studies done to date (6). Despite being a point of intense research, the critical question of how to prevent, stop, or revert the aggregation of α-synuclein remains unresolved.

α-Synuclein is a 140-amino acid protein with little ordered structure that binds to lipid membranes. It comprises seven repeats, in the positively charged amino-terminal region and the hydrophobic middle part, with the carboxyl-terminal region being negatively charged. Monomeric α-synuclein adopts an α-helical structure upon binding to lipid membranes containing acidic phospholipids. This conformation involves amino acids 1–98, with residues 99–140 being considered unstructured (7).

In recent years, the mechanism of α-synuclein aggregation has been compared with that of the prion protein, whose misfolding causes transmissible spongiform encephalopathies (8). Common biochemical hallmarks are the propensity to aggregate, insolubility in mild detergents, and partial resistance to protease digestion (9–11). The development and use of protein misfolding cyclic amplification (PMCA)^3^ of the prion protein (12) has helped to understand the underlying prion replication, infectivity, and strain formation.

## EXPERIMENTAL PROCEDURES

### 

#### 

##### Expression and Purification of Recombinant Wild-type α-Synuclein

BL21(DE3) *Escherichia coli* was transformed with human full-length α-synuclein in pRK172, and the protein was then purified as described (13). Briefly, bacterial cells were harvested and resuspended in Tris/EDTA buffer, lysed 4 °C (with 25 kg/square inch using a cell disruptor (Constant Systems Ltd.) and centrifuged). α-Synuclein protein was purified from the lysate supernatant by anion exchange using HiTrap Capto adhere (GE Healthcare), (NH_4_)_2_SO_4_ precipitation, gel filtration, and anion exchange using Mono Q GL (GE Healthcare). The pooled protein fractions collected from the purification steps were concentrated and solvent-exchanged using Amicon Ultra-15 centrifugal filters with 10-kDa molecular mass cutoff (Millipore). Aliquots of protein were stored at −20 °C prior to use. A 10-μl aliquot was hydrolyzed in 6 m HCl for amino acid analysis. Protein concentrations were determined by quantitative amino acid analysis, performed in-house (LMB-MRC, UK), and confirmed at the Protein and Nucleic Acid Chemistry Facility, University of Cambridge, UK.

##### PMCA

PMCA was carried out by subjecting recombinant wild-type full-length human α-synuclein to repeated cycles of sonication and incubation. α-Synuclein was prepared as indicated (13) and diluted to a final 90 μm concentration in conversion buffer (1% Triton X-100, 150 mm NaCl, Complete Protease Inhibitor Mixture (Roche Applied Science; in 1×PBS). For PMCA, 60-μl aliquots from 200 μl of the 90 μm reaction mixtures were transferred into 200-μl PCR tubes (Axygen) containing 37 ± 3 mg of 1.0-mm zirconia/silica beads (Biospec Products), and samples were subjected to cycles of 20-s sonication and 30-min incubation at 37 °C, for different times depending on the experiment, using a Misonix 4000 sonicator at 70 power setting. All reactions were performed in triplicate. When drugs or seeds were used, 2 μl of concentrated drugs were added into 200 μl of the PMCA reaction mixture. Seeded reactions (for the study of substrate concentrations and the serial PMCA) were done by diluting 1:100 of 90 μm α-synuclein fibrils, previously generated by PMCA, into fresh soluble α-synuclein recombinant substrate.

##### Thioflavin T Assay

From each sample, 5 μl was added to 495 μl of ThT solution (20 μm ThT, 50 mm glycine in H_2_O, pH 8.5, with KOH). Fluorescence was measured with a PerkinElmer Life Sciences luminescence spectrophotometer LSS5 with 450-nm excitation and 480-nm emission settings.

##### Far-UV Circular Dichroism Spectroscopy (CD)

Conformational changes in α-synuclein PMCA samples were monitored using a CD spectrometer (Jasco J-810), taking an average of five scans at 100 nm/min over the spectral range of 190–260 nm. The samples, first tested for ThT fluorescence, were loaded into a 0.5-mm path length quartz cuvette (Hellma) and scanned in Peltier temperature-controlled unit (Jasco), at 20 °C. The CD spectrum of the buffer alone was also evaluated and found to produce negligible spectra. The relative increase in secondary structure, corresponding to α-synuclein aggregation, was determined based on the decrease in negative absorbance, with a peak ∼200 nm and subsequent simultaneous increases in negative absorbance with a peak ∼ 218 nm, consistent with a change of structure from disordered monomers to β-sheet-rich amyloid fibrils.

##### Transmission Electron Microscopy

The morphology of α-synuclein aggregates in PMCA samples was examined by transmission electron microscopy using a Phillips model EM208S microscope operated at 80 keV. Three-μl aliquots of 24 h PMCA or 8-day incubated samples were placed directly on carbon-coated 400-mesh grids, briefly washed with ddH_2_O, and negatively stained with 1–2% (w/v) phosphotungstic acid. Observations were made over a wide range of magnifications up to ×110,000 using a built-in CCD camera.

##### Native and SDS Gels

Three-μl aliquots of α-synuclein PMCA or non-PMCA control samples were either mixed with 1 μl of 4× loading buffer (NuPAGE LDS®; Invitrogen) and incubated at 100 °C for 10 min (for SDS gels) or mixed with 1 μl of 4× native loading buffer (NativePAGE®; Invitrogen), and 3.5 μl of the mixture was loaded into 4–12% SDS (Bis-Tris)- or native gels (Invitrogen). Either low molecular mass standard (Bio-Rad) or SeeBlue Plus2 (Invitrogen) protein ladders were used as molecular mass markers for Bis-Tris gels, whereas NativeMark® unstained protein standards were used for native gels. In some cases gels were stained using only Coomassie Blue, whereas in other experiments proteins were transferred onto PVDF Immobilon membranes (Millipore), and α-synuclein was visualized by incubation with monoclonal or polyclonal anti-α-synuclein antibodies. Chemiluminescence was induced by ECL-Plus (Pierce) and recorded with the Alliance software (Uvitec Cambridge).

##### Proteinase K (PK) Digestion

Aliquots of 20 μl of α-synuclein PMCA samples or controls (non-PMCA) were incubated for 30 min at 37 °C with 4 μl of 0, 15, 60, or 600 μg/ml PK (Roche Applied Science) in conversion buffer with a final concentration of 0, 2.5, 10, or 100 μg/ml PK. Enzymatic reactions were terminated by adding 6 μl of 4× loading buffer (NuPAGE LDS®) and heating for 10 min at 95 °C. Fifteen-μl samples were loaded onto 4–12% or 12% Bis-Tris gels, and SeeBlue Plus2 was used as molecular mass standard. Gels were either stained with Coomassie Blue or electrotransferred for immunoblotting.

##### α-Synuclein PMCA “Defibrillation”

Drugs were added to 90 μm α-synuclein fibrils, previously generated by PMCA in a final 50 μm concentration and incubated at 37 °C with agitation at 750 rpm for 30 min. Samples were cooled, and 5 μl was taken to determine the presence of α-synuclein fibrils using the ThT assay as described previously.

##### Drugs and Antibodies

Congo red, curcumin, quinacrine, resveratrol, lacmoid acid, tannic acid, ibuprofen, acetaminophen, and aspirin (all Sigma) were diluted in DMSO at various concentrations and then diluted also in DMSO at 1 and 5 mm concentrations. Of these aliquots, 2 μl were added to 198 μl of PMCA reactions for final 10 and 50 μm concentrations. The anti-α-synuclein antibodies Syn1 (BD Biosciences), 5C2 (Novus Biologicals), Per7 (14) and Per4 (3) were used for immunoblotting. The antibody Syn1 and Hoechst 33342 dye were used for immunofluorescence. The epitopes of the anti-α-synuclein antibody are: Per7, 1–120; 5C2, 61–95; LB509, 115–122; Per4, carboxyl-terminal; Syn1, 91–99.

##### Cell Infection with α-Synuclein PMCA Fibrils

SH-SY5Y (5 × 10^5^) cells stably overexpressing human full-length α-synuclein (15) were seeded with either sonicated α-synuclein PMCA fibrils or monomeric recombinant α-synuclein (used as control) at a 3 μg/ml concentration in the cell media. Confluent cells were split 4 days after infection, and in every following passage a cell aliquot was plated on glass coverslips and immunostained fluorescently to detect α-synuclein aggregates. Ten fields per sample were counted in three different experiments.

## RESULTS

### 

#### 

##### Establishment of a Reproducible and Sensitive Method to Produce α-Synuclein Aggregates

**FIGURE 1.**
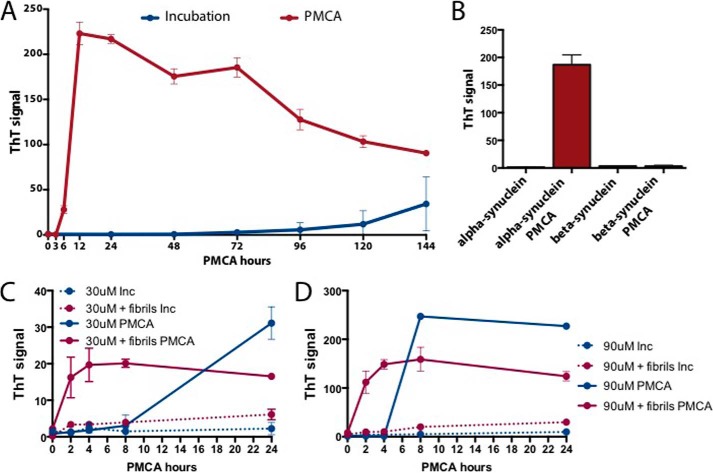
**α-Synuclein PMCA.** *A*, growth kinetics of full-length recombinant α-synuclein fibril assembly by PMCA and incubation methods. *B*, α- and β-synuclein PMCA fibril formation compared with non-PMCA samples in a 24-h reaction. *C* and *D*, kinetics of α-synuclein fibril formation with PMCA or incubation (*Inc*) using 30 μm (*C*) or 90 μm (*D*) recombinant α-synuclein substrate, with or without seeding with recombinant α-synuclein PMCA fibrils. Assembly was monitored by the enhancement over time of ThT fluorescence intensity at 480 nm. Each point represents mean ± S.D. (*error bars*) of three replicates and is representative of two experiments. Fibril formation is faster with PMCA compared with the incubation method both in the presence and absence of α-synuclein fibril seeds.

In view of the propensity of α-synuclein to aggregate *in vitro* we set out to establish an α-synuclein PMCA to generate recombinant wild-type α-synuclein fibril assembly. The PMCA technique combines cycles of incubation at 37 °C (to grow fibrils) and sonication (to break fibrils into smaller growing fractions) of samples containing Triton X-100 for solubility, avoiding precipitation of the aggregates. We compared the kinetics of full-length α-synuclein fibril growth by PMCA with the traditional incubation/shaking method, over 8 days and at nine different time points. ThT 480-nm emission was used as the readout for fibril assembly (16). The results (Fig. 1*A*) show that PMCA induces a faster kinetic of filamentous aggregate formation compared with incubation and shaking. Fibril formation was detected following 6 h of PMCA with the maximal signal reached between 12 and 24 h following the beginning of the reaction. By the time α-synuclein fibrils were obtained by PMCA, and using the same concentration of recombinant protein, no fibrils were seen with the incubation/shaking method. With the latter method some ThT signal was observed after 4 days but with high variability. Furthermore, no ThT signal was present when PMCA was performed using β- instead of α-synuclein (Fig. 1*B*). After 24/48 h there was a decline in the PMCA-induced ThT signal. To clarify the reasons for this decrease the biochemical characteristics of the samples at 1 and 8 days following PMCA were studied by SDS-PAGE with Coomassie Blue staining and Western blotting. The results showed that following 8 days of PMCA no monomeric α-synuclein was detectable, and all of the protein was concentrated in a high molecular mass smear (data not shown). It is likely that ThT does not have easy access to big aggregates, as those present in our system at this time point, and hence the reduced signal, although more work is needed to confirm this hypothesis.

To explore the effect of seeding on the initial substrate concentration and time required for fibril formation, we investigated the kinetics of fibril formation of 30 and 90 μm (Fig. 1, *C* and *D*) recombinant α-synuclein substrates in the absence or presence of 0.9 μm α-synuclein fibrils in a 24-h PMCA or in a regular incubation reaction. When recombinant α-synuclein fibrils were added to the reaction at both concentrations, a small increase in the ThT signal was observed with the incubation method, whereas using PMCA fibrils formed as fast as 2 h, with the maximum level reached between 4 and 8 h (Fig. 1, *C* and *D*). The results of the 24-h 90 μm PMCA samples shown (Fig. 1, *A*, *B*, and *D*), have a ThT signal average of 210.3 ± 20.4 with a 9.7% standard deviation, exhibiting high reproducibility between experiments.

##### Biochemical Characterization of α-Synuclein Fibrils Generated by PMCA

**FIGURE 2.**
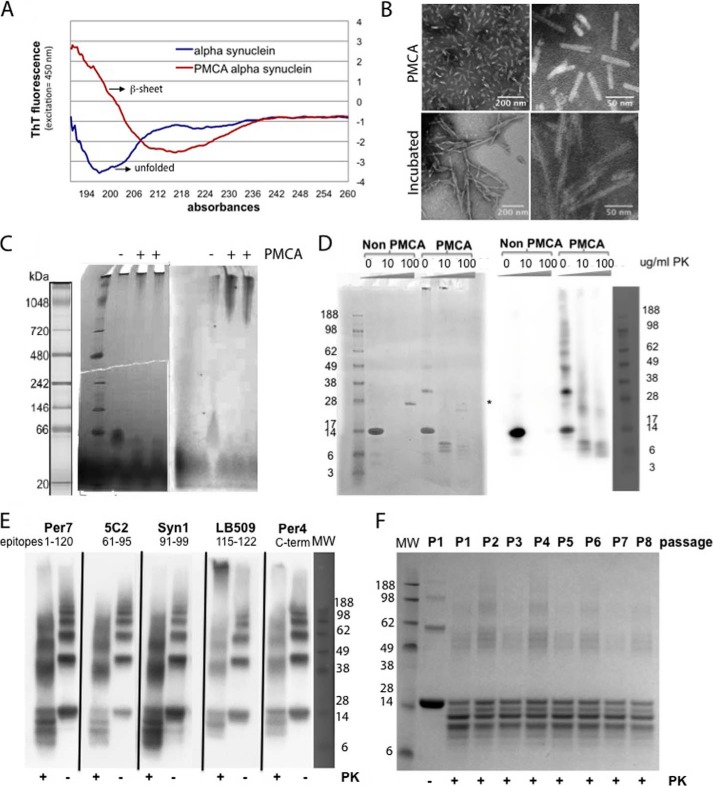
**Characterization of α-synuclein PMCA-derived fibrils.** *A*, circular dichroism of recombinant α-synuclein before and after 24-h PMCA. *B*, transmission electron microscopy of 24-h PMCA (*upper panels*) and incubation (*lower panels*) α-synuclein fibrils in carbon coated grids at two different magnifications. *C*, Coomassie Blue-stained (*left*) and anti-α-synuclein fluorescence-immunostained (*right*) native gels of recombinant α-synuclein subjected (+) or not (−) to 24-h PMCA. An increase in high molecular mass species is present following PMCA. *D*, Coomassie Blue-stained (*left*) or anti-α-synuclein immunostained (*right*) Bis-Tris gel before and after 24-h PMCA samples following digestion with different concentrations of PK. Whereas non-PMCA samples contain mainly monomeric protein that is completely degraded by PK, the PMCA-derived samples show specific bands following PK digestion. *Asterisk* indicates the position of the PK band in the Coomassie Blue-stained gel. *E*, α-synuclein 24-h PMCA, before (−) and after (+) PK digestion, epitope mapping using several anti-α-synuclein-specific antibodies. *F*, Coomassie Blue-stained SDS (Bis-Tris) gel of serial α-synuclein 24-h PMCA samples after 2.5 μg/ml PK digestion. No clear significant difference is observed in band pattern after PK digestion in samples from different PMCA passages.

The biochemical characteristics of the α-synuclein material generated by PMCA were investigated by several techniques to confirm aggregate formation. Circular dichroism (CD) was performed to compare the product of PMCA α-synuclein and non-PMCA control samples (Fig. 2*A*). Comparison of the spectra showed an increase in β-sheet content in α-synuclein PMCA samples compared with the non-PMCA-treated α-synuclein that remained mainly unfolded. Negative staining electron microscopy was performed on the samples. Fibrils of heterogeneous sizes were present in high amounts in the PMCA α-synuclein sample reaction whereas in the incubated sample filaments were less abundant and longer (Fig. 2*B*). Native gel electrophoresis (Fig. 2*C*) was also used to compare samples subjected or not to 24-h PMCA. Both Coomassie Blue staining and immunoblotting with specific α-synuclein antibodies showed that only after PMCA were large aggregates of α-synuclein present. The proteinase K resistance of recombinant α-synuclein PMCA samples compared with non-PMCA samples was then determined (Fig. 2*D*). Non-PMCA-treated protein was easily digested by 10 μg/ml PK whereas the α-synuclein PMCA sample was resistant to digestion up to 100 μg/ml PK. Immunoblotting of 2.5 μg/ml PK-digested samples with antibodies against five α-synuclein epitopes located in the amino-terminal, central, and carboxyl-terminal part of the protein was performed. The result suggested that the amino-terminal fragment of the protein was resistant to PK digestion (Fig. 2*E*). Finally the existence of “strain-like” modifications in the fibril formation by performing serial PMCAs was investigated, but the pattern of bands after PK digestion remained constant after eight passages (Fig. 2*F*).

##### α-Synuclein PMCA for Anti-amyloid Drug Testing

Our aim was to set up a rapid system for screening compounds affecting α-synuclein aggregation. We therefore investigated the effects on PMCA aggregation of α-synuclein of compounds previously described to affect amyloid aggregation differently.

Congo red and curcumin were selected because their effect has been widely studied in prions and they have been also reported to interact with α-synuclein filamentous aggregates (17–19). As a negative control quinacrine was selected because it is known not to inhibit prion aggregation *in vitro* although it is effective *in vivo* (20). Other previously studied drugs, non-steroidal anti-inflammatory drugs such as Ibuprofen (21), acetaminophen (22), and aspirin (23) were used to evaluate the specificity of the assay. The remaining drugs tested included lacmoid and resveratrol, with reported binding to α-synuclein (24, 25); tannic acid and (−)epigallocatechin gallate (EGCG), previously described as potential inhibitors of α-synuclein aggregation (26–29).

**FIGURE 3.**
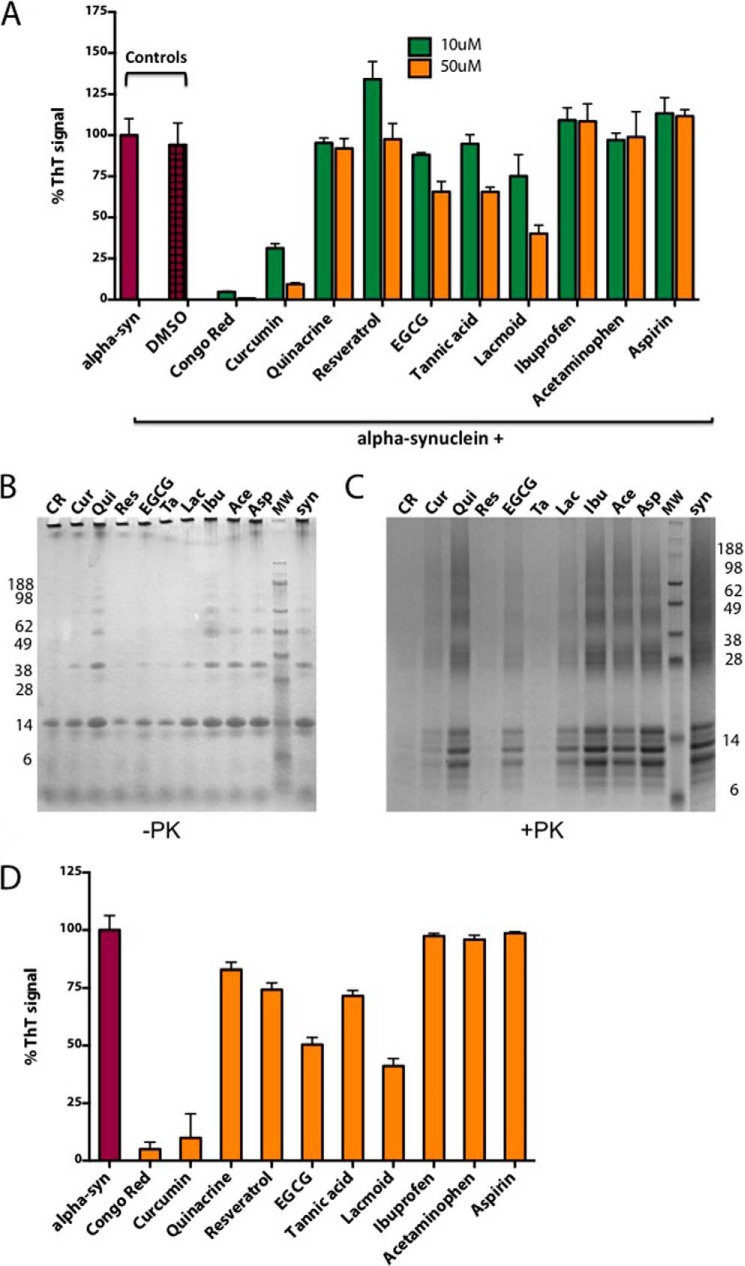
**Compound screening using α-synuclein PMCA.** *A*, 16-h α-synuclein (*alpha-syn*) PMCA reaction, alone or in the presence of solvent (DMSO) and two concentrations of 10 different drugs added at the beginning of the reaction. ThT signal is measured as readout for α-synuclein fibril assembly. Data are normalized for α-synuclein signal (α-synuclein) without drugs and presented as mean ± S.D. (*error bars*) of triplicate samples in two independent experiments. *B* and *C*, Coomassie Blue staining of SDS (Bis-Tris) gel of the drug-PMCA samples before (*B*) and after (*C*) digestion with 2.5 μg/ml PK. The order of the drugs (as indicated by name abbreviation) corresponds to that in *A*. The *lanes* indicated as *syn* show α-synuclein without drugs. *D*, ThT signal of defibrillated α-synuclein (*alpha-syn*) PMCA samples following 30-min incubation with 50 μm concentration of 10 different drugs added to preformed fibrils.

We initially established that DMSO, used to dilute the drugs, did not affect α-synuclein PMCA, then we tested two different concentrations of each drug, 10 and 50 μm, to determine presence and potency of their inhibition in a 16-h PMCA reaction. The drug screening results (Fig. 3*A*) showed a great percentage of inhibition of ThT signal (70–90%) by Congo red and curcumin at both concentrations tested, lower inhibition (35–40%) with EGCG, tannic acid, and lacmoid, and no inhibition with the remaining drugs. To avoid artifacts, the results were confirmed by SDS-PAGE before and after PK digestion followed by Coomassie Blue staining. This revealed that also resveratrol (which has fluorescence emission in the presence of β-sheet structures that overlaps with the emission of ThT (25)) was inhibiting α-synuclein aggregation during PMCA (Fig. 3*B*). Furthermore, we studied the disaggregating properties of the same battery of drugs in preformed PMCA α-synuclein fibrils. The same drugs that inhibited the aggregation of α-synuclein during PMCA (Fig. 3*C*) were also able to disaggregate preformed α-synuclein aggregates.

##### α-Synuclein “Chronically Infected” Cells

**FIGURE 4.**
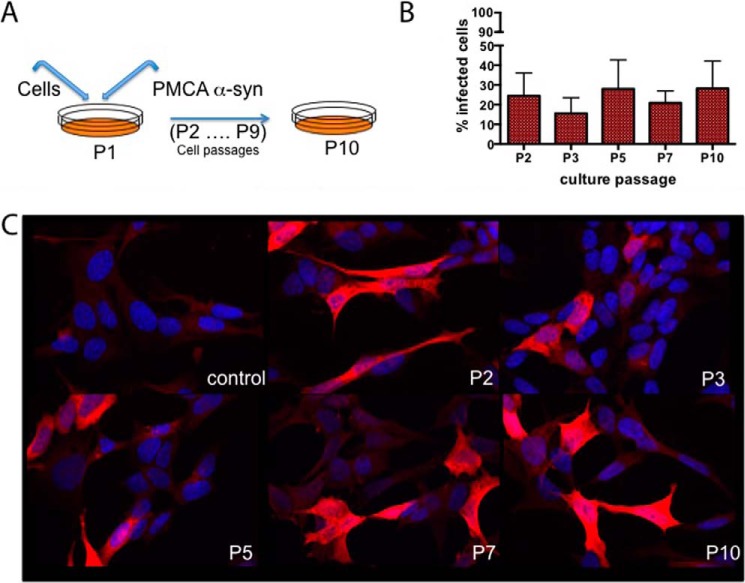
**α-Synuclein aggregation cell model.** *A*, diagram representing the cell “infection” experiment using SH-SY5Y neuroblastoma cells overexpressing human α-synuclein. *B*, cell count expressed as percentage of infected cells (showing accumulated α-synuclein) per passage. These results are from three independent experiments. *C*, immunofluorescence staining using Syn1 anti-α-synuclein antibody (*red*) and Hoechst dye staining (*blue*) in α-synuclein-transfected SH-SY5Y control cells and cells collected at several passages after incubation with PMCA-derived aggregates.

To confirm the efficacy of the compounds selected by PMCA, we set up a cellular system from which to obtain α-synuclein aggregates. SH-SY5Y neuroblastoma cells stably transfected with human full-length α-synuclein were exposed to α-synuclein PMCA material and then split on confluence (Fig. 4*A*). The presence of α-synuclein aggregates was investigated by immunofluorescence with anti-α-synuclein antibodies, and the result showed accumulation of α-synuclein for up to 10 divisions following the initial exposure to the aggregates (Fig. 4*C*). The percentage of cells with accumulated α-synuclein remained fairly constant at approximately 25% during the passages, and without further exposure, indicating that the cells were chronically infected (Fig. 4*B*).

## DISCUSSION

α-Synuclein is a critical protein in Parkinson disease and other neurodegenerative diseases called α-synucleinopathies. Several missense mutations, duplications and triplications of the α-synuclein gene (*SNCA*) are associated with hereditary forms of Parkinson disease. Additionally, all sporadic Parkinson cases as well as those associated with α-synuclein mutations have aggregated α-synuclein in Lewy bodies. These aggregates are believed to be involved in toxicity and contribute to the loss of neuronal function (for review, see Ref. 30). Moreover, the ability of α-synuclein aggregates to travel from cell to cell, spreading as seeds to form newly misfolded α-synuclein aggregates in host cells (31–33), makes the protein a target for therapy. Indeed, inhibiting the aggregation of α-synuclein would impede cell-to-cell transmission of seeds and stop progression of the disease.

Recombinant α-synuclein can form *in vitro* filamentous aggregates similar to those found in human brain (32–35). Although recombinant α-synuclein aggregates have been formed *in vitro* for 15 years, the methods used have some limitations mainly for two reasons: unless mutant α-synuclein is used, the generation of fibrils takes days and the concentration of recombinant protein needed is very high, between 300 and 500 μm. Recently, an α-synuclein-adapted PMCA was published (36), but it still required high concentration of recombinant protein, seeding of the reaction with preformed fibrils and long times.

In this study we have set up a highly efficient PMCA for α-synuclein based on modifications of prion PMCA (12). In a concentration-dependent reaction we can generate α-synuclein fibrils in 6 h, or 2 h when seeds of preformed fibrils are added. The concentration of recombinant α-synuclein is much lower than in most methods described previously (37, 38) for aggregation; moreover, the method is highly reproducible.

When α-synuclein fibril formation rate was compared between PMCA and the traditional incubation method a significant difference was found. Fibrils were present already after 6 h of PMCA whereas using the incubation method and the same low substrate concentration, filaments only started to appear after 4 days. Furthermore, the PMCA method showed great reproducibility between the triplicates or between different experiments as indicated by the low S.D. values, averaging 10% S.D. both within the same experiment and between different experiments, whereas this was not the case for the incubation method that had greater variability.

To study whether the α-synuclein aggregation was specific and not an artifact of a system that would generate aggregates out of any protein, we performed PMCA using as substrate β-synuclein. This protein has a 63% homology with α-synuclein, and is not present in Lewy body filaments. Furthermore, it does not aggregate *in vitro* unless in the presence of metals, glycosaminoglycans, or molecular crowding (39). The result demonstrated that the PMCA was specific for α-synuclein, as an aggregation prone protein, because no fibrils were obtained when β-synuclein was used.

*In vitro* generated α-synuclein aggregates have been shown to present biophysical and biochemical characteristics similar to *in vivo* α-synuclein aggregates, and therefore we wanted to determine whether our PMCA-generated α-synuclein aggregates had the same hallmarks. The folding pattern of α-synuclein after PMCA revealed a high content in β-sheet structure by circular dichroism compared with the predominantly unfolded non-PMCA control. Electron microscopy and negative staining showed in the PMCA samples a heterogeneous population of fibrils with different lengths, in contrast to the longer filaments obtained with the incubation method. The difference in the fibril length is probably a reflection of the breakup of the PMCA α-synuclein fibrils during sonication. When the PMCA samples were run on native gels they showed high molecular mass aggregates compared with the low molecular mass forms of the soluble native protein in the non-PMCA control. As for the resistance of newly generated α-synuclein aggregates to digestion with proteinase K, there were fragments still resistant to high concentrations of the enzyme in the PMCA sample compared with the non-PMCA control. Those fragments consisted mainly of amino-terminal regions, as anti-α-synuclein antibodies recognizing epitopes toward the carboxyl terminus, such as LB509 and Per4, failed to recognize some of the PK-resistant fragments. The unchanged pattern of PK-resistant bands in a serial PMCA indicated an absence of conformational or “strain” differences (40) in the samples.

Together, these results show that α-synuclein PMCA promotes the formation of α-synuclein aggregates with all biochemical features characteristic of α-synuclein aggregates *in vivo*. Therefore, the α-synuclein PMCA is a fast and low protein consuming method to mimic α-synuclein fibril growth. Thereafter, we explored the potential of the α-synuclein PMCA to screen for compounds that by interfering with α-synuclein aggregation would be candidates for therapy in α-synucleinopathies.

A panel of 10 compounds was chosen to prove our concept. The anti-amyloid properties of some compounds (Congo red, curcumin, resveratrol) previously established for aggregation-prone proteins, such as prions, β-amyloid, or α-synuclein. Non-steroidal anti-inflammatory drugs, initially described as α-synuclein aggregate modulators (23), but without real effect in patients, as demonstrated by epidemiological studies (41, 42), were included to test PMCA specificity. Other compounds were reported to bind α-synuclein or to alter its aggregation in other *in vitro* assays. Finally, quinacrine did not have any relationship with amyloids or protein aggregation and therefore was chosen as a negative control. In our drug screening α-synuclein aggregation was strongly inhibited by Congo red, curcumin, and resveratrol (as shown by PK digestion but not ThT assay for the latter, because resveratrol has its own fluorescence), and to a lower extent by EGCG, tannic acid, and lacmoid. Very recently it was reported that lacmoid does not prevent α-synuclein aggregation as measured by “amyloid intrinsic fluorescence” (43); however, here we showed by Coomassie Blue and Western blotting that the content of high molecular α-synuclein bands and PK resistance were decreased following lacmoid. This confirmed that the decrease in ThT labeling in the presence of lacmoid corresponded in fact to a reduction of aggregated α-synuclein. None of the non-steroidal anti-inflammatory drugs influenced the formation of α-synuclein aggregates. These results, where only the drugs with proven anti-amyloid activity and/or interaction with α-synuclein have an effect inhibiting the reaction, revealed a high specificity of the system in detecting drugs with a high probability to interfere with α-synuclein pathology. Moreover, PMCA was done in a high-throughput format that will allow screening of a large number of compounds using low amounts of recombinant proteins and in short time. Therefore we believe that the α-synuclein PMCA model that we present here is a good tool for anti-aggregation drug screening.

To further extend the α-synuclein fibril characterization, looking at spreading, and to generate an *ex vivo* assay for further drug screening, we inoculated SH-SY5Y neuroblastoma cells overexpressing human full-length α-synuclein with α-synuclein PMCA fibrils. Although the cells were in contact with the recombinant fibrils for just 4 days at the beginning of the experiment, a constant percentage of infected cells (cells with accumulated α-synuclein) was present at all analyzed cell passages. We hypothesize that probably there are two mechanisms involved in the maintenance of the persistent infection: first, the turnover of cells dying and other naïve cells taking up the released aggregates, and secondly the existence of cell-to-cell transmission. Further studies to verify our hypothesis are currently ongoing. Nevertheless, these chronically infected cultures provide an expandable and reproducible cellular system for α-synuclein aggregation that can be used for drug testing, as well as for investigating the pathways involved in the spread and cell response to α-synuclein aggregation.

In summary, our results show that α-synuclein PMCA is a fast and reproducible system that can be used for high-throughput screening for α-synuclein anti-aggregating compounds. This system, complemented with chronically infected cells, is relevant for identifying therapeutic compounds for Parkinson disease and other α-synucleinopathies.

## Footnotes

3 The abbreviations used are:

PMCA: protein misfolding cyclic amplification
Bis-Tris: bis(2-hydroxyethyl)iminotris(hydroxymethyl)methane
DMSO: dimethyl sulfoxide
EGCG: (−)epigallocatechin gallate
PK: proteinase K
ThT: thioflavin T.
